# An Expanded Self-Antigen Peptidome Is Carried by the Human Lymph As Compared to the Plasma

**DOI:** 10.1371/journal.pone.0009863

**Published:** 2010-03-26

**Authors:** Cristina C. Clement, Elvira S. Cannizzo, Maria-Dorothea Nastke, Ranjit Sahu, Waldemar Olszewski, Norman E. Miller, Lawrence J. Stern, Laura Santambrogio

**Affiliations:** 1 Department of Pathology, Albert Einstein College of Medicine, New York, New York, United States of America; 2 Department of Pathology, University of Massachusetts Medical School, Worcester, Massachusetts, United States of America; 3 Department of Surgical Research and Transplantology, Polish Academy of Sciences, Warsaw, Poland; 4 Magdalen College, University of Oxford, Oxford, United Kingdom; 5 Department of Microbiology and Immunology, Albert Einstein College of Medicine, New York, New York, United States of America; Agency for Science, Technology and Research (A*STAR), Singapore

## Abstract

**Background:**

The pre-nodal afferent lymph is the fluid which directly derives from the extracellular milieu from every parenchymal organ and, as it continues to circulate between the cells, it collects products deriving from the organ metabolism/catabolism. A comprehensive qualitative and quantitative investigation of the self-antigenic repertoire transported by the human lymph is still missing.

**Methodology/Principal Findings:**

A major difference between lymph and plasma could be visualized by FPLC and 2D gel in the amount of low molecular weight products corresponding to peptide fragments. Naturally processed peptides in normal pre-nodal human lymph were then fractionated by HPLC and characterized by multidimensional mass spectrometry. Analysis of more then 300 sequences identified self-peptides derived from both intracellular and extracellular proteins revealing the variety of catabolic products transported by human lymph. Quantitative analysis established that at least some of these peptides are present in the circulating lymph in nanomolar concentration.

**Conclusions/Significance:**

The peptidome, generated by physiological tissue catabolism and transported by the pre-nodal lymph, is in addition to the self-peptidome generated in endosomal compartment. Unlike self antigen processed by local or nodal APC, which mostly produce epitopes constrained by the endosomal processing activity, self antigens present in the lymph could derived from a wider variety of processing pathways; including caspases, involved in cellular apoptosis, and ADAM and other metalloproteinases involved in surface receptor editing, cytokines processing and matrix remodeling. Altogether, expanding the tissue-specific self-repertoire available for the maintenance of immunological tolerance.

## Introduction

Four mechanisms are likely to guarantee processing and presentation of a wide variety of tissue-specific self antigens: (i) self proteins are phagocytosed and processed in peripheral tissue by local antigen presenting cells and displayed to T cells patrolling peripheral organs [1]; (ii) products of “self” are carried through the lymphatic system by circulating dendritic cells (DC) [2]–[6]; (iii) lymph nodal cells expressing AIRE encode tissue-specific proteins, similarly to thymic epithelial cells [7], [8]; (iv) self antigens are transported from the parenchymal tissue to the draining lymph nodes, through the lymphatic system [9], [10]. The first three mechanisms rely on antigen processing and presentation by parenchymal and nodal APC, which mostly produce an MHC class II peptidome restricted by endosomal proteases, among which cathepsins have been characterized in greatest details [11]. The last mechanism is the least characterized among the four, mostly due to the great difficulty to obtain primary lymph material. As such, a comprehensive qualitative and quantitative investigation of the self-antigenic repertoire transported by the lymph is still missing.

Lymphatic fluid (lymph) is derived from the tissue fluid compartment and constitutes up to 20% of the body weight [12]. Four different types of lymph have been classified: 1) interstitial lymph that is enclosed in the intercellular spaces throughout the body; 2) circulating lymph that circulates through the lymphatic vessels towards the veins; 3) chyle, the circulating lymph collected from the intestinal epithelia during digestion; and 4) serous lymph, the liquids normally contained in the pleural, peritoneal and pericardial cavities, in the cerebral ventricles, and the cerebro-spinal fluid. The tissue lymph is the fluid which directly derives from the extracellular milieu from every parenchymal organ, and as it continues to circulate between the cells, it collects products deriving from the organ metabolism/catabolism [13]. In order to be transported to the lymph nodes, the lymph is then collected into lymphatic capillaries, which form a mesh-like network of blind-end tubes distributed throughout the tissue spaces. The capillaries flow into progressively larger lymphatic vessels that transport the pre-nodal lymph to the 400–500 lymph nodes disseminated throughout the human body [14]. The lymph enters through the cortical area of the node and by traveling through the conduit system in the inter follicular T cell areas conveys a representative sampling of the interstitial fluid to the nodal antigen presenting cells before entering the central vein located in the nodal sinus [14]–[17].

In general it has always been assumed that the lymph would contain a qualitatively similar protein composition as the plasma. A previously published partial comparative proteomic analysis between bovine lymph and plasma indicated indeed an almost overlapping proteomic between the two samples with only few proteins being differentially expressed [12]. As in plasma, the most abundant proteins where identified as albumin, immunoglobulins, transferrin, fibrinogen and apolipoproteins [12]. However, differently from the plasma, the lymph directly collects from the extracellular milieu of each parenchymal organ; thus it could potentially be a much richer source of peripherally derived tissue specific antigens. Indeed, experiments of cellular and extracellular *“in vivo”* radioactive labeling as well as cellular tracking have indicated that products of cellular apoptosis and extracellular matrix turnover are found in the lymph [18], [19].

The primary hypothesis that initiated this investigation was that the lymph could potentially carry a wider antigenic repertoire than the plasma and be a richer source of tissue specific antigens. Additionally, we also hypothesized that, differently from plasma; the lymph could carry a partially processed proteome/peptidome since it directly collects from the extracellular milieu where products of tissue catabolism, tissue remodeling, cellular apoptosis, and extracellular matrix processing are collected before being transported to the draining lymph nodes.

In this work we present an in depth MS/MS and quantitative analysis of the pre-nodal human lymph, which does indeed indicate an expanded self-antigen repertoire carried by the human lymph as compared to plasma from the same subjects. The proteome/peptidome, generated by physiological tissue catabolism, and transported by the lymph is additional to the self-peptidome generated in endosomal compartment; altogether expanding the tissue-specific self-repertoire available for the maintenance of immunological tolerance.

## Results

### Self-Peptidomic Analysis of the Human Lymph

**Figure 1:**
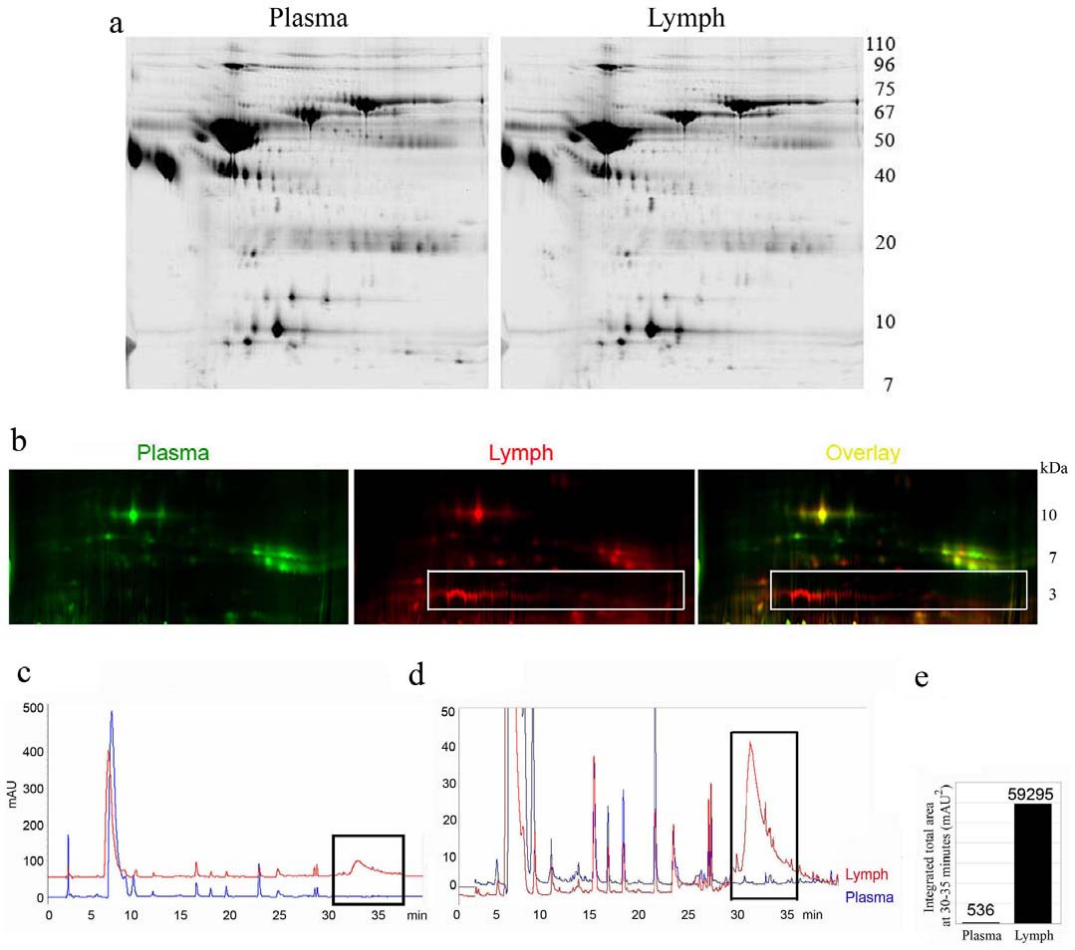
Comparative peptidomic profile of human lymph and plasma samples. **a**) Comparative 2D-gel analysis of human lymph and plasma proteins (2CyDye). **b**) Fluorescence (2CyDye) comparative 2D-gel analysis of human lymph and plasma low molecular weight products (1–10 kDa). One out of 6 silver staining gels is shown **c**) Overlays of reverse phase HPLC profile of lymph and plasma low MW filtrates (<5000 Da). Samples from 18 subjects were pooled. **d**) is a 10 times magnification of **c**. Boxed areas indicate where major differences between the two samples are observed. One out of four reverse phase HPLC analysis is shown. **e**) Bar graph representing the integration number calculated from the curves in the boxed area for both the lymph and the plasma pooled samples.

A previously published proteomic analysis of the bovine lymph identified several major proteins common to both lymph and plasma (albumin, fibrinogen, IgG, transferrin, lactoferrin and apolipoproteins) and altogether indicated a qualitatively similar protein profile between the two samples [12]–[13]. Complementary to this analysis we performed a comparative analysis between human lymph and subject-matched plasma pooled from 18 individuals (Figure 1a). Since the presence of very abundant proteins could interfere with the analysis, samples were depleted of albumin and IgG prior to 2D gel analysis. An almost overlapping proteomic profile could be observed between the two samples; however, an observed major difference between lymph and plasma could be visualized in the 2D gel in the amount of low MW products (Figure 1b). Similarly, comparative reverse phase HPLC analysis of lymph and plasma samples indicated substantial overlap between the two (Figure 1c and 1d) except for a broad peak eluting at around 30–40 minutes (Figure 1c, 1d boxed area). This peak had a 100-fold greater intensity in the lymph as compared to the plasma (Figure 1e).

**Figure 2:**
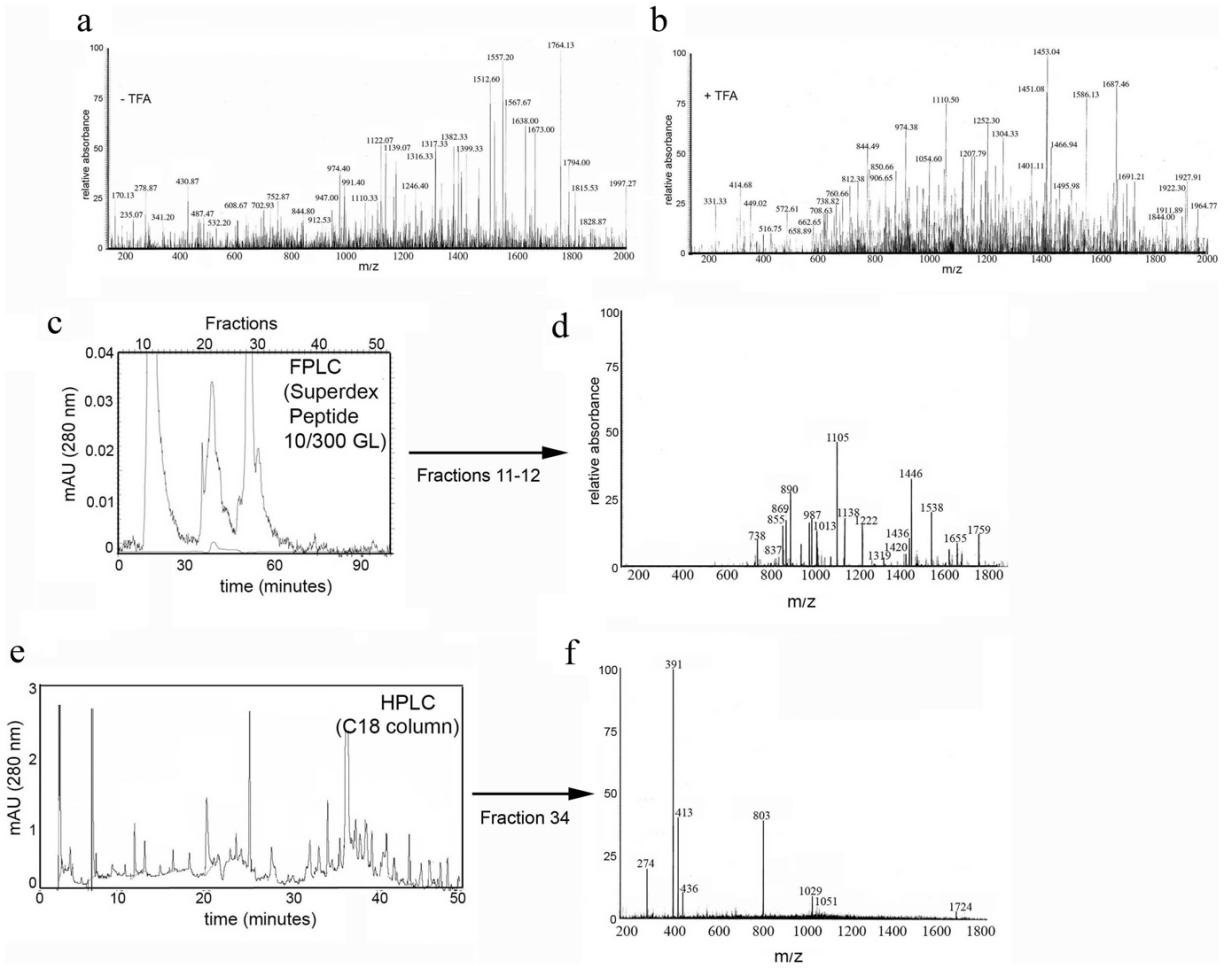
MS peptidomic profile of the human lymph. **a and b**) Mass spectroscopy scan (200–2000 m/z) of low MW (5000 Da) filtrate from lymph samples obtained before or after elution in 0.1% TFA. **c**) Elution profile of the low MW filtrate (<5000 Da) collected from the lymph pooled samples run through a superdex peptide 10/300 GL column **d**) MS scan of eluted fractions 11–12. **e**) Representative elution profile of the low MW filtrate (<5000 Da) collected from the lymph pooled samples run through a C18 column **f**) Representative full MS scan of eluted fraction 34.

To further analyze the low molecular weight species present in the human lymph, samples of pooled lymph (from 18 subjects) were treated in the presence or absence of 0.1% TFA to release any additional peptide possibly bound to serum and plasma proteins (Figure 2a, 2b), before <5000 MW cutoff ultrafiltration and multidimensional mass spectrometry scan (200–2000 m/z) analysis. A significant pool of peptides was present in both untreated (Figure 2a) and TFA-treated (Figure 2b) samples. To facilitate MS/MS identification of each molecular species, eluted peptides were first either fractionated by FPLC on a Superdex peptide gel-filtration column (Figure 2c) or by HPLC, on a C18 reverse-phase, column (Figure 2e). Each fraction was then analyzed by tandem MS/MS using Nanospray LC-MS/MS on a LTQ linear ion trap mass spectrometer (Figure 2d and 2f). The tandem MS/MS identified more than 300 peptides using MASCOT and SeqQuest algorithms and non-redundant NCBI and SwissProt databases (see Methods for details).

**Figure 3:**
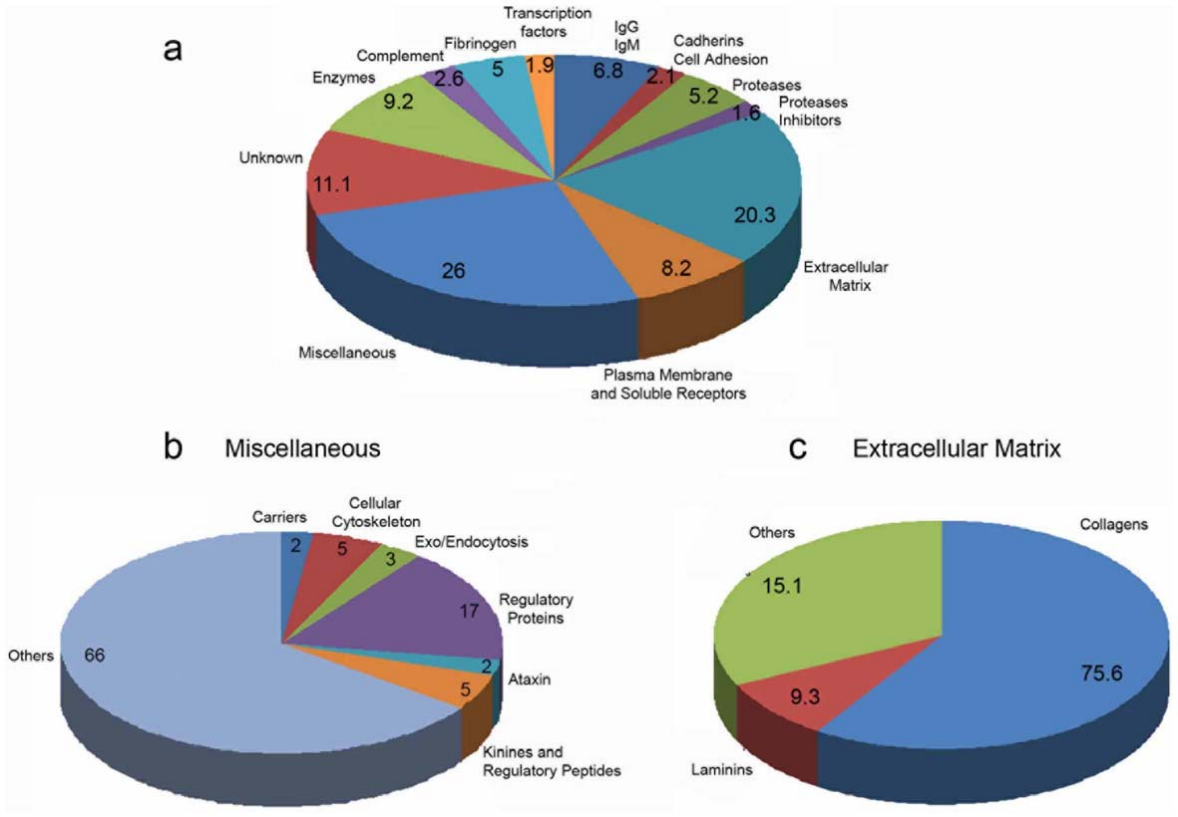
Schematic representation of the MS/MS peptidomic profile of the human lymph. **a, b and c**) Pie chart representation of self-peptides (<5000 Da) sequenced from the human lymph. Data are compiled from 4 independent MS/MS analysis.

The identified self-peptides derived from both intracellular and extracellular proteins revealing the variety of catabolic products transported by human lymph (Figure 3 and Figure S1). A large number of the sequenced peptides derive from the immunoglobulin family (6.8%), complement (2.6%) and fibrinogen (5%). Several peptides derived from processed plasma membrane-associated or soluble receptors (8.2%) as well as cadherins and cell adhesion molecules (2.1%) (Figure 3a and Figure S1). A major category of sequenced peptides derived from processing of extracellular matrix proteins (20.3%). Many others (26%) were categorized as miscellaneous and a relatively large pool (11.1%) was represented by peptides of unknown origin (Figure 3 and Figure S1). A breakdown analysis of the miscellaneous peptide pool indicated the presence of peptides derived from processing of proteins from the cytoskeleton, exo/endocytosis and several regulatory proteins together with kinines and derived regulatory peptides (Figure 3b). In addition, the statistical analysis of the extracellular matrix peptide pool revealed collagens as a dominant population (75.6%), followed by laminins (9.3%) (Figure 3c). Altogether the analysis indicates that the majority of sequenced peptides derive from catabolic processing of extracellular or plasma membrane bound proteins (Figure 3 and Figure S1).

### Many peptides carried by the human lymph are derived from the degradation of extracellular matrix proteins

**Figure 4:**
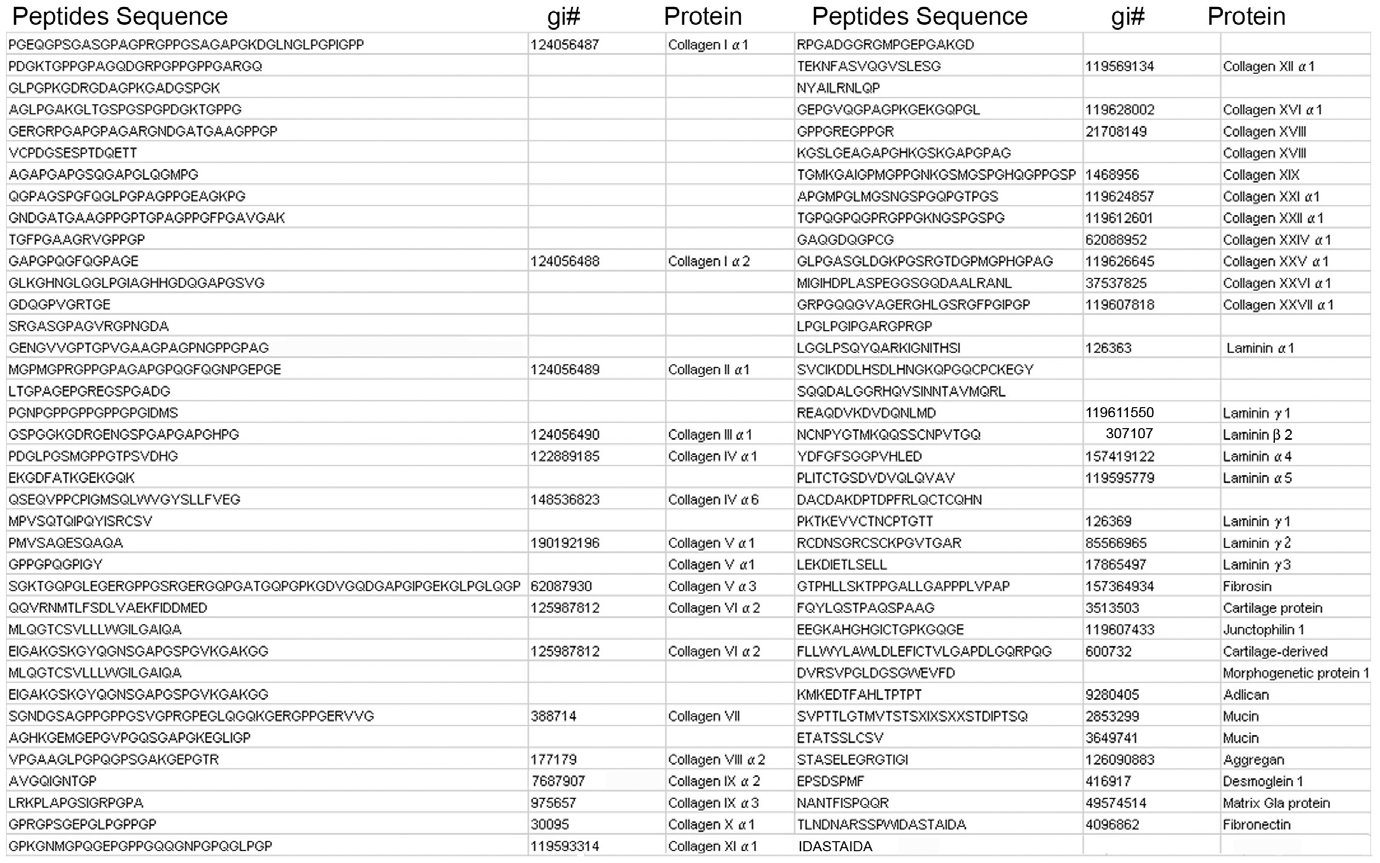
Amino acid sequence of self-peptides derived from processing of extracellular matrix proteins found in the human lymph. MS/MS sequencing analysis of peptides found in the human lymph, which derive from processing of extracellular matrix proteins.

Since a large number of peptides retrieved from human lymph derived from extracellular matrix components, we further focused our study on the detailed analysis of the self-peptidome of the human extracellular matrix. More than 70 peptides were sequenced and almost all classes of collagens were found. Peptides derived from laminin alpha1, alpha4, alpha5, gamma 1–3 were also found in the proportion described in Figure 3c. Less represented were peptides derived from fibrosin, cartilage protein, adlican, mucins and fibronectin catabolism (Figure 4).

**Figure 5:**
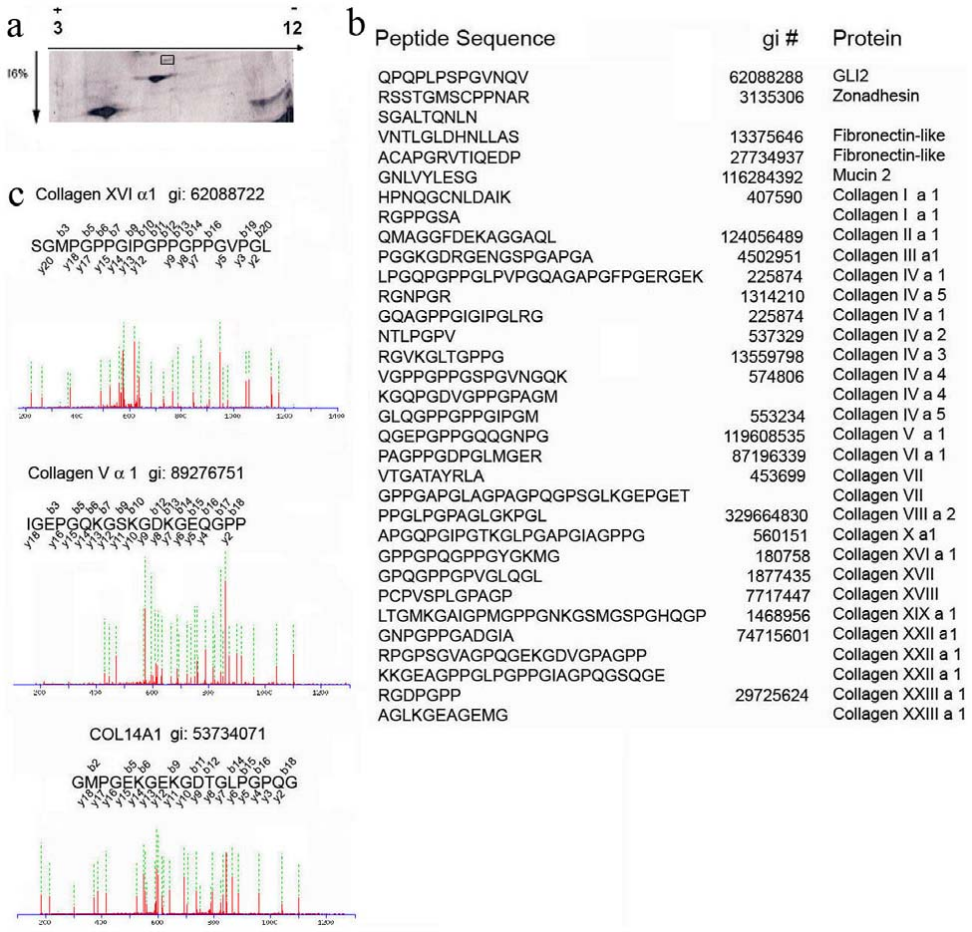
Quantification of 2D gel eluted peptide. **a**) Silver staining of an SDS-PAGE run with 20 microgram of immunoglobulin and albumin depleted lymph. **b**) Sequence of some of the peptides retrieved from each of the analyzed spot. **c**) MS/MS fragmentation profile and respective sequence of the three peptides eluted from spot number 6. Green dotted lines identified assigned fragmentation picks. Total peptide concentration eluted from spot number 6 calculated at 230 OD was 0.22 µM.

The large number of peptides derived from extracellular matrix turnover prompted us to address the issue of whether these peptides were present in a quantifiable amount. To determine this total lymph, previously depleted of immunoglobulin and albumin, was run on a 2D 16% SDS-PAGE gel to resolve the low molecular weight peptide species. Silver staining analysis identified several spots around and below 3000 kDa (Figure 5a). Single spots were extracted from the gel and analyzed by LTQ-MS/MS coupled with database search using MASCOT algorithm (Figure 5b). A significant amount of the peptides (16% of the total number of sequenced peptides) were derived from extracellular matrix proteins, with the highest population represented by the collagens (Figure 5b and data not shown).

To quantify the amount of peptides present in a single 2D spot we selected spot number 6 for gel extraction and LTQ-MS/MS analysis. Three peptides were sequenced from spot number 6 (Figure 5c). Visualization of silver stained proteins is dependent on both total amount and physical characteristics (amino acid primary sequence and overall peptide length) of the protein; however, in general silver staining can visualize an amount of protein in the low nanogram range. For short peptides, a much greater amount is needed to be visible in silver staining. The relative absorbance of the three combined gel-extracted peptides was determined at 230 nm (extinction coefficient 300 M-1 cm-1 per peptide bond), assuming an average length of 19 residues (18 peptide bonds), an extinction coefficient of 5,400 was determined. Altogether the three gel eluted peptides were quantified to be around 0.22 µm. A more quantitative measurement was performed on the material eluted from spot number 6 by quantitative amino acid analysis which indicated a total peptides concentration of 180 ng (data not shown). Altogether, the 2D quantitative analysis of peptides derived from collagens and other extracellular matrix antigens established that at least some of these peptides are present in the circulating lymph in nanomolar concentration. Peptide therapies for tolerance induction have been previously shown to be effective even at sub-nanomolar peptide concentration.

### HLA-DR-1 and HLA-DR-4 MHC class II binding and immune recognition of extracellular Matrix Peptides

Next we determined whether any of the extracellular matrix fragments sequenced from the human lymph were able to bind to MHC II molecules. We tested HLA-DR1 and HLA-DR4 molecules since many autoimmune diseases with self reactivity to extracellular matrix proteins are HLA-DR1 or HLA-DR4 associated [22]–[23]. A positive control peptide, previously reported to represent a bona fide extracellular matrix autoantigen presented by HLA-DR1 and HLA-DR4 [21], was included in the analysis (AGFKGEQGPKGEP from Collagen I α1, IC50 1.5 uM).

**Figure 6:**
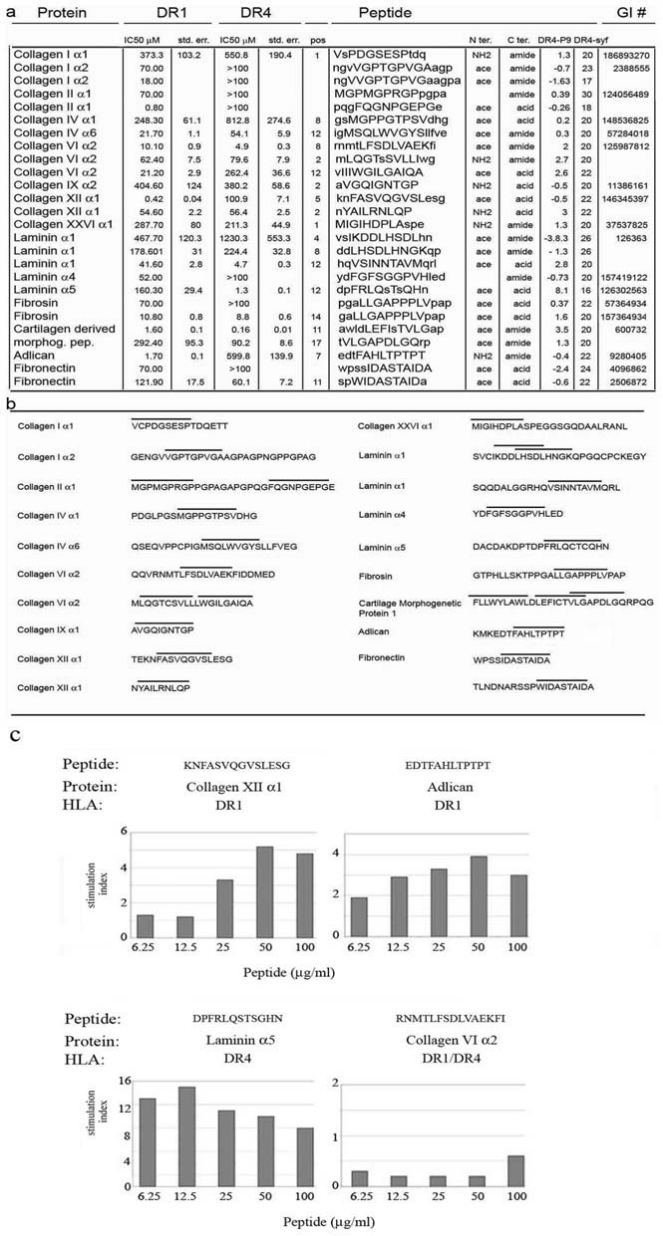
HLA-DR1 and HLA-DR4 binding affinity and T cell proliferative response to some of the lymph sequenced peptides. **a**) IC50, as determined by competitive fluorescence polarization assay, for short peptides derived from fragments identified in lymph. Peptides were selected based on predicted binding of nonameric binding frames (capital letters) to HLA-DR1 or HLA-DR4. Peptides were synthesized with N-terminal acetyl and C-terminal amide groups unless the peptide termini were coincident with an end of the sequenced fragment, in which case the native terminus was used **b**) Position of the assayed peptides in the sequenced fragments indicated by bars above the sequence. **c**) Proliferative responses (calculated by thymidine incorporation assay) in lymph nodes from peptide immunized mice. One out of four proliferation assay is shown for each peptide.

We identified potential HLA-DR1 or HLA-DR4 binding regions within the extracellular matrix fragment sequences from human lymph, using an efficient prediction algorithm [21]. For each potential nonameric binding frame, we synthesized a 12–15 residue peptide containing the region of interest in its natural context, and determined the binding affinity using a competition fluorescence polarization assay (Figure 6a). Several peptides binding with affinity comparable to the test autoantigens were identified, including peptides from collagen II α1, collagen XII α1, cartilage-derived morphogenic protein, and adlican for HLA-DR1 and collagen VI α2, laminin α1, laminin α5, and fibronectin for HLA-DR4 (Figure 6b).

Finally we asked whether any of the tight-binding peptides, which also were conserved between mouse and human, could be recognized by peripheral T cells. HLA-DR1 or HLA-DR4 transgenic mice were immunized with 100 µg of several of the extracellular matrix peptides in complete Freund's adjuvant in the flanks and nape of the neck. Draining lymph nodes were collected 14 days later and reactive T cells analyzed by proliferation assay with thymidine incorporation. A significant proliferation index was observed in response to some of the immunizing antigens (Collagen XII, Adlican and Laminin α5). The result indicates that autoreactive T cells specific to these antigens are present in the peripheral T cell repertoire (Figure 6c).

## Discussion

We report here a comparative analysis of the overall self antigenic repertoire present in the human lymph as compared to plasma from matching donors. We confirmed as previously established that the overall proteomics between lymph and plasma is very similar with only few proteins uniquely expressed in the lymph but not in the plasma [12], [13]. Unexpectedly, however, we also find that a major difference between lymph and plasma is in the peptidome profile. Whereas very few peptides are found in the plasma, a much greater amount of protein fragments and short peptides are present in the lymph.

Sequence analysis of more then 300 peptides indicates that processed fragments derived from both intracellular and extracellular proteins. When the peptidome profile described here was compared to previously published MHC class II eluted peptidome, it appeared that the lymph peptide repertoire is partially skewed towards peptides derived from extracellular matrix proteins, cadherins and cleavable or soluble receptors (43%) [24], [25].

Some of the peptides we identified in the human lymph were derived from intracellular proteins. These are likely byproducts from apoptotic cells. In physiological cell catabolism, older differentiated cells are mostly removed by apoptosis and replaced by the progeny of tissue adult stem cells [26]–[30]. Apoptotic cells are mostly phagocytosed “in situ” by tissue macrophages and dendritic cells; however, some apoptotic cells have also been found in the circulating lymph [19]. Thus, partially processed proteins and peptides from intracellular origins found in the human lymph are likely released by the dying cells [19].

On the other hand, lymph-carried peptides derived from the catabolism of the extracellular matrix likely reflect the constant remodeling of the extracellular connective tissue [18], [31]. The extracellular matrix components constitute a structure that helps to maintain tissue integrity, regulates cell growth, division and migration, and provides a reservoir of cytokines, chemokines and growth factors [32]–[35]. By its very nature, the extra-cellular matrix is constantly undergoing changes and it has been previously estimated that collagen in the skin and in parenchymal organs has a very rapid turnover (10–30% day). The turnover is accelerated in response to various cellular stimuli which range from dynamic homeostasis to full-blown tissue remodeling, as occurs during inflammation, wound healing, and cancer [35].

Data from the sequencing analysis prompted us to ask whether the self antigens peptide fragments, generated by physiological tissue catabolism, found in the human lymph would play a role towards the maintenance of peripheral tolerance. From an immunological standpoint for the lymph carried peptides to be tolerogenic their amount should be in a quantitative relevant amount. To this end we compared the amount of peptides found in our analysis with the amount previously determined to be effective is several peptide-based therapeutic vaccines aimed at recovery of immunological tolerance [36]–[38]. Peptide therapy have been shown to reduce immune responses to allergens and down-regulate cytokines and T cell proliferative responses in different diseases among which asthma, rheumatoid arthritis, type 1 diabetes and cat and bee allergy [39]–[43]. Mice experimental work indicate dose ranges between few micrograms to milligrams, human clinical trials report effectiveness of few micrograms of subcutaneously injected peptides in the treatment of asthmatic individuals [36]. Peptide concentration it is recognized to be important in inducing low versus high tolerance. It is generally accepted that high peptides regiments induce tolerance by depleting auto-reactive antigen specific T cells as well as inducing regulatory T cells; on the other hand low peptides regiments only appear to induce regulatory T cells [36]. Low dose tolerance is induced upon injection of sub-immunogenic doses of soluble antigens and therapeutical efficacy has been demonstrated even with sub-nanomolar doses of antigens [37]. In our analysis we determined that several peptides could be visualized on a 2D gel and estimated to be in high-nanomolar concentration. Importantly, peptides directly found in the lymphatic fluid are readily and constantly available to nodal antigen presenting cells. In experimental immunization and in peptide-therapy clinical trials one dose of peptide is administered weekly or even monthly either subcutaneously or intradermally and no data are available on determining the actual peptide concentration reaching the blood and the lymph [36].

With regard to the APC population targeted by the lymph- carried peptides, it has been previously established that subcutaneously injected antigens are transported to the lymph node sub-cortical macrophages and by the conduit system to the interfollicular DC [10]. It is likely that lymph-transported peptides are also delivered to the immature DC associated with the conduit system. Once in the lymph node, peptides could load directly at the cell surface of immature DC as previously reported, either on empty MHC class II molecules or by exchanging with previously loaded peptides at the plasma membrane or in early recycling compartments [44], [45]. On the other hand, protein fragments and longer peptides are more likely to be internalized for MHC class II loading in endosomal multivesicular bodies [46] or following the cross presentation pathway for MHC class I loading [47].

In conclusion even though it has often been hypothesized that the lymph would collect material from parenchymal cell and extracellular matrix catabolism, this is the first analysis that actually proves that an immunologically relevant amount of proteins, partially processed proteins and peptides are carried by the human lymph. The extracellular peptidome describe herein likely reflect the protein composition of the tissues (skin, adipose tissue, connective and muscle) represented at the site of lymph collection. In the future it would be of interest to compare pre-nodal lymph collected from capillaries draining different tissues to determine a tissue specific proteome/peptidome. Unlike self antigen processed by local or nodal APC, which mostly produce epitopes constrained by the endosomal processing activity, self antigens present in the lymph could derived from a wider variety of processing pathways; including caspases, involved in cellular apoptosis, and ADAM and other metalloproteinases involved in surface receptor editing, cytokines processing and matrix remodeling. Altogether, broadening the sample of peptides available to endosomal and plasma membrane MHC class II loading.

## Materials and Methods

### Collection of pre-nodal lymph and plasma

#### Subjects

Eighteen healthy males aged 22 to 36 years were studied. All had been given a full medical examination, and screened by EKG and laboratory examination to exclude cardiovascular, liver, renal, endocrine and hematologic disease, and recreational drug abuse. None was taking a special diet or medication. The study had been approved by the Royal London School of Medicine Committee for Clinical Investigation, and all subjects gave informed written consent. The Albert Einstein College of Medicine, Committee for Clinical Investigation, approved the analysis on human lymph and plasma. Alcohol was avoided for 48 hours before and during the study.

#### Clinical Procedures

Prenodal peripheral lymph was collected as previously described [20]. Subjects were admitted to a metabolic ward 24 or 48 hours before the collection, and placed on an isocaloric diet. Lymph vessel cannulations were per formed between 8:00 and 11:00 a.m in a surgical theater under full sterile conditions. The subjects had been fasted overnight, but allowed unlimited access to fluids. The lower part of one leg was shaved to about 20 cm above the ankle, and the skin sterilized with 0.05% chlorhexidene. An area of skin (4 cm^2^) 6–10 cm above the ankle over the anteromedial aspect was anesthetized with subcutaneous 2% lignocaine in 1∶100,000 epinephrine. A horizontal 15–20 mm incision was made in this area. Under an operating microscope, the subcutaneous lymph vessels were dissected, and one was selected for cannulation. A second small incision (3 mm) was made 10 mm above the first, through which a cannula (Intramedic® polyethylene tubing PE60; Becton Dickinson & Company, Sparks, MD; cat. no. 427416; i.d. 0.76 mm, o.d. 1.22 mm) was passed. The cannula was inserted into the vessel for 5–10 mm (towards the foot), and secured with a silk ligature (Mersilk, 5/0). The other (untapered) end of the cannula was passed into a 2-mL screw-topped polypropylene cryovial (Nunc A/S, Roskilde, Denmark). A blood sample from each subject was also drawn. Blood and lymph samples were centrifuged at 1,500 *g* for 15 min at 4°C, and the supernatants transferred to polypropylene microcentrifuge tubes [20]. Samples were supplemented with a cocktail of proteases inhibitors (Roche).

### 2D DIGE Protein Expression Profiling of human lymph and plasma

Equal amounts of protein from whole lymph and plasma (pooled from 18 patients) (between 2 and 6 mg/sample) were depleted of immunoglobulins and albumin using the Aurum Serum protein kit (Biorad). Fifty ug of each sample was subjected to fluorescence labeling using 2 dyes (Cy3 for plasma and Cy5 for lymph) and to 2D-DIGE preparative electrophoresis (Applied BIOMICS Inc. facilities, Hayward, CA). After electrophoresis, the gel was scanned using a Typhoon image scanner and the images were analyzed using the DeCyder software. Protein spots of interest were automatically picked from the 2D gel with the Ettan Spot Picker, subject to tryptic digestion and MS/MS analysis.

### Fractionation of lymph and plasma for mass spectroscopy analysis

An equal amount of proteins from both plasma and lymph (10 mg) were filtrated through Centriplus (Amicon) centrifugal filter devices (5,000 Da cut-off). A cocktail of protease inhibitors (Roche) was added to each sample to avoid proteolysis. The filtrate was quantified by UV measurements at 230 nm (for determination of peptide concentration). In an independent experiment, samples (10 mg) were treated with trifluoroacetic acid (TFA) (at 0.1% final concentration in the sample) to extract the peptides adsorbed on different proteins, chaperones and albumin. Peptide sample filtrates were then lyophilized, resuspended in 0.1% TFA and subjected to desalting using ZipTip (C18) reversed-phase system (Millipore, Billerica, MA) or PepClean C-18 spin columns (Pierce).

### High pressure liquid chromatography (HPLC) and gel-filtration chromatography of plasma and lymph filtrates

Plasma and lymph (5,000 kDa filtrates) were separated using a reversed-phase system (buffer A: 0.1% TFA and buffer B: 0.1% TFA in acetonitrile) on a C18 column on a Hewlett Packard HPLC system (1100 series). Alternatively, plasma and lymph filtrates were run through a Superdex peptide 10/300 gel filtration column (Tricorn) in 30% acetonitrile in 0.1% TFA as solvent. Each fraction was lyophilized before mass spectroscopy analysis.

### LTQ-tandem MS/MS sequencing of peptides from lymph filtrates

LTQ-MS/MS sequencing of peptides present in the lymph (5,000 kDa filtrates) or from HPLC or gel-filtration fractions was performed using Nanospray LC-MS/MS on a LTQ linear ion trap mass spectrometer (LTQ, Thermo, San Jose, CA) interfaced with a TriVersa NanoMate nanoelectrospray ion source (Advion BioSciences, Ithaca, NY). MS/MS was performed using an isolation width of 2 *m/z*, normalized collision energy of 35% and a minimum signal intensity of 1000 counts. The sequences of each peptide were determined using the LTQ software analysis MS (mass spectroscopy) database searching with MASCOT (Mowse score above the predicted identity score) and SEQUEST (Delta Cn above 0.15, Sp200 percentage ions above 80 and Xscorr above 2) algorithms). The MS/MS scans were screened against NCBInr database as a first choice and then validated against SWISSPROT database.

### Fluorescence polarization Peptide binding assays

To evaluate the binding affinities of peptides to HLA-DR1 (HLA-DRA*0101,-DRB1*0101) and HLA-DR4 (HLA-DRA*0101,-DRB1*0401) molecules a competition assay was performed in a 96-well setting. The assay is based on the differential fluorescence polarization of MHC-bound and free test peptide acetyl-PRYVKQNTLRLAT, derived from influenza hemagglutinin 306–318 and labeled at the internal lysine residue using the amine-reactive reagent Alexa488-tetrafluorophenyl ester (Invitrogen). Serial dilutions of unlabeled peptide competitors (∼10 uM to ∼30 nM) were incubated with Alexa488-HA peptide (25 nM) and with either HLA-DR1 (100 nM) or a mixture of HLA-DR4 and HLA-DM (100 nM each). HLA proteins were prepared as soluble extracellular domains expressed in insect cells as described [21]. Unlabeled HA peptide was used as a control competitor. MHC-peptide mixtures was incubated for 3 days at 37°C in 100 mM sodium citrate (pH 5)/50 mM sodium chloride buffer (pH 5.5), containing 0.25% octylglucoside, 185 ug/ml iodoacetamide (IAA), 25 nM EDTA, 0.1% NaN_3_ and freshly added 0.5 ug/ml PMSF. For HLA-DM incubations 1 mM DTT was added instead of IAA. Polarization measurements were performed in a POLARstar OPTIMA plate reader (BMG Labtech), using the fluorescence polarization detection mode and 485 nm excitation and 520 nm emission filters. Fluorescence polarization values for free and MHC-bound Alexa-HA peptide were 70 and 350 mP, respectively. IC_50_ values were evaluated using a competitive binding equation in GraphPadPrism, and are reported as mean and standard error for single fit to triplicate experimental values. IC_50_ value for the control unlabelled HA peptides was 25 nM.

### 2D-electrophoresis of proteins from whole human lymph

For 2-D analysis of whole lymph, the lymph sample (10 mg protein) was pre-cleaned using the 2-D cleaning kit from Biorad. Aliquots of pre-cleaned 50 ug protein sample were further resuspended in 2D-EF loading buffer containing 7 M urea, 2 M thiourea, CHAPS (4%), NDSB 256 (5%), protease cocktail inhibitors (Roche), 10 mM Tris, pH 8.0 and IPG buffer (0.5%) (pH 3–10 NL from GE Healthcare). Samples were loaded on an Immobiline DryStrip, pH 3–12, NL, 13 cm, Biorad and run for isoelectric focusing for the first dimension. Strips were rehydrated for a total of 14,934 Vhours. The second dimension was conducted on a 4–10% SDS-PAGE protein gel or on a 10–20% Tris/Tricine/SDS peptide gel. The gels were stained using standard procedures with silver stain (SilverSNAP stain kit, Pierce). For tandem MS/MS sequencing of peptides with MW<5000 Da, the peptides were extracted from the gel in a solution containing 50% acetonitrile and 0.1% TFA, at 37°C, overnight. The extracted peptides were further analyzed in tandem MS/MS mode using LTQ, ESI (+) mode as described above.

### Peptide Immunization, T cell Proliferation

HLA-DR1 and HLA-DR4 mice were immunized in the flanks and nape of the neck with 100 micrograms of peptide emulsion in Freund's adjuvant. Two weeks later popliteal and axillary lymph nodes were harvested and tested in proliferation assay. Briefly 6×10^5^ cells were seeded in a 96 well plate with or without titrated amount of the immunizing peptide for 72 hours. Thymidine (1 µCu) was added 18 hours before cell harvesting. Cells were harvested on a Tomtec harvester (Model 94-3-468) and incorporated thymidine counted on a liquid scintillation counter (1450 Microbeta Wallac Trilux).

## Supporting Information

Figure S1 Peptidomic profile of the human lymph. MS/MS sequencing analysis of peptides found in the human lymph.
(0.02 MB DOC)
