# Supplementary material for: An Expanded Self-Antigen Peptidome Is Carried by the Human Lymph As Compared to the Plasma
Source: PLoS One. 2010 Mar 26;5(3):e9863. doi: 10.1371/journal.pone.0009863 (PMC2845622; doi:10.1371/journal.pone.0009863)
Supplement: Figure S1 — Peptidomic profile of the human lymph. MS/MS sequencing analysis of peptides found in the human lymph. (0.02 MB DOC) [file pone.0009863.s001.doc]

**Figure S1: Peptidomic profile of the human lymph.**

MS/MS sequencing analysis of peptides found in the human lymph
